# Molecular Mechanisms and Pathways in Visceral Pain

**DOI:** 10.3390/cells14151146

**Published:** 2025-07-25

**Authors:** Qiqi Zhou, George Nicholas Verne

**Affiliations:** 1 College of Medicine, University of Tennessee, Memphis, TN 38163, USA; gverne@uthsc.edu; 2 Lt. Col. Luke Weathers, Jr. VA Medical Center, Memphis, TN 38105, USA

**Keywords:** disorders of gut–brain interaction, irritable bowel syndrome, visceral hypersensitivity, ncRNA

## Abstract

Chronic visceral pain, a significant contributor to morbidity in the United States, affects millions and results in substantial economic costs. Despite its impact, the mechanisms underlying disorders of gut–brain interaction (DGBIs), such as irritable bowel syndrome (IBS), remain poorly understood. Visceral hypersensitivity, a hallmark of chronic visceral pain, involves an enhanced pain response in internal organs to normal stimuli. Various factors like inflammation, intestinal hyperpermeability, and epigenetic modifications influence its presentation. Emerging evidence suggests that persistent colonic stimuli, disrupted gut barriers, and altered non-coding RNA (ncRNA) expression contribute to the pathophysiology of visceral pain. Additionally, cross-sensitization of afferent pathways shared by pelvic organs underpins the overlap of chronic pelvic pain disorders, such as interstitial cystitis and IBS. Central sensitization and viscerosomatic convergence further exacerbate pain, with evidence showing IBS patients exhibit hypersensitivity to both visceral and somatic stimuli. The molecular mechanisms of visceral pain involve critical mediators such as cytokines, prostaglandins, and neuropeptides, alongside ion channels like transient receptor potential vanilloid 1 (TRPV1) and acid-sensing ion channels (ASICs). These molecular insights indicate potential therapeutic targets and highlight the possible use of TRPV1 antagonists and ASIC inhibitors to mitigate visceral pain. This review explores the neurophysiological pathways of visceral pain, focusing on peripheral and central sensitization mechanisms, to advance the development of targeted treatments for chronic pain syndromes, particularly IBS and related disorders.

## 1. Chronic Visceral Pain: Mechanisms and Overlapping Pathologies

Chronic visceral pain is among the most debilitating functional disorders, impacting an estimated 100 million individuals in the United States. This condition, which underlies disorders of gut–brain interaction (DGBIs) such as irritable bowel syndrome (IBS), imposes significant healthcare and economic burdens, with costs exceeding USD 700 billion annually. Beyond the financial implications, the condition dramatically impairs the quality of life, manifesting in persistent discomfort, emotional distress, and reduced productivity. Despite its widespread prevalence and severe repercussions, the underlying mechanisms driving DGBIs and chronic visceral pain remain poorly understood, posing challenges to the development of effective therapeutic interventions [1,2,3,4].

While significant strides have been made in understanding DGBIs, the pathophysiology of visceral pain remains elusive [5]. Several mechanisms may contribute to this condition, including neuroinflammation, peripheral sensitization, and gut barrier dysfunction. Neuroinflammation is thought to exacerbate pain signals by altering neural pathways, while peripheral sensitization increases the responsiveness of nociceptors to stimuli. Meanwhile, gut barrier dysfunction may facilitate the translocation of antigens and pathogens that trigger immune responses and sensitize visceral nerves [6]. However, these mechanisms are often studied in isolation, and the field still lacks an integrated framework that cohesively explains their interactions. Addressing this gap is crucial for advancing targeted therapies and improving outcomes for individuals living with these chronic conditions.

This review aims to explore the molecular and physiological underpinnings of visceral pain, emphasizing the neurophysiological pathways and the contributions of central and peripheral sensitization. By bridging basic science with clinical implications, it seeks to provide a comprehensive perspective on the mechanisms of visceral pain, laying the groundwork for the development of targeted and effective therapeutic strategies. Advancing our understanding of these mechanisms is essential for addressing the unmet needs of patients and improving their quality of life.

## 2. Pathophysiology of Visceral Pain and Hypersensitivity

Chronic abdominal pain, which is the leading cause of visits to gastroenterology clinics, is most commonly attributed to DGBIs, particularly IBS [7]. While IBS is characterized by abdominal pain associated with altered bowel habits, it is also frequently accompanied by an exaggerated pain response to otherwise normal stimuli in the gut and other systems [4], known as visceral hypersensitivity or hyperalgesia. In these cases, even mild stimuli (e.g., distention or mild contractions) are perceived as intensely painful, a phenomenon often accompanied by allodynia (pain in response to non-painful stimuli). This extended response goes beyond the gastrointestinal (GI) tract, implicating multiple interconnected pathways. Heightened sensitivity represents a maladaptive neural response to environmental factors, previous injury, or stress, and highlights the complex interaction between peripheral mechanisms, such as those driven by gut inflammation, and central mechanisms within the nervous system.

Emerging evidence has identified several critical mechanisms that contribute to visceral hypersensitivity and pain, each contributing to a multi-level interaction between the peripheral and central nervous systems:

1. Neuronal sensitization: Persistent, abnormal stimuli from the colon can lead to prolonged hypersensitivity by sensitizing spinal neurons [8,9]. This process involves viscerosomatic convergence, where nociceptive input from visceral structures (e.g., the gut and other internal organs) overlaps with somatic input (e.g., from the skin, muscles, and soft tissues), leading to a compound response. The sustained input from the colon can “prime” spinal neurons, making them more responsive to future stimuli [9].
2. Increased intestinal permeability: The gut barrier is crucial for maintaining immune homeostasis and protecting against harmful stimuli [10]. However, barrier disruptions, such as those seen in conditions like IBS, can contribute to pain by allowing pathogenic or inflammatory mediators (e.g., cytokines, bacteria) to interact with afferent nerve fibers [6]. This “leaky gut” phenomenon may not only heighten nociception but also activate immune responses that further sensitize visceral pathways [10].
3. Epigenetic influences: Recent studies have illuminated the role of epigenetic regulation in the pathophysiology of visceral pain [11]. Altered expression of microRNAs (miRNAs) in GI tissues, potentially delivered via extracellular vesicles (EVs), may affect the expression of pain-related genes. These small RNA molecules can modulate pain signaling pathways at both the peripheral and central levels, adding another layer of complexity to visceral hypersensitivity mechanisms.

These factors demonstrate the dynamic interplay between peripheral and central mechanisms and underscore the importance of studying both to fully understand chronic visceral pain [12]. This dual-layered process, involving both the gut and the brain, challenges current therapeutic approaches that often focus on only one aspect while excluding the other.

### 2.1. Mechanisms of Neuronal Sensitization

Ongoing research continues to unravel the complexities of visceral pain mechanisms, with a growing focus on peripheral and central sensitization. The interplay between these processes underscores the need for a comprehensive approach that addresses the full spectrum of factors contributing to chronic pain. The roles of silent nociceptors, immune–neural interactions, and epigenetic modifications are among the emerging areas of interest, offering a more nuanced understanding of visceral hypersensitivity and chronic pain syndromes.

#### 2.1.1. Afferent Mechanisms of Visceral Pain

Primary visceral afferents in the gut are pivotal in the development and maintenance of chronic visceral hypersensitivity, as shown by studies in both human and animal models [13]. These specialized sensory neurons convey stimuli from the gut to the central nervous system (CNS), forming the basis for gut sensory processing [2]. Their receptors are distributed across the serosal, muscular, and mucosal layers of the GI tract, allowing them to detect mechanical (e.g., distension), chemical, and luminal stimuli. While most GI input remains below conscious perception, pathological triggers such as inflammation, trauma, or environmental stressors can sensitize the gut, heightening its response to luminal distension. This sensitization underlies the visceral hypersensitivity observed in IBS patients, contributing to symptoms such as bloating, abdominal pain, and altered bowel habits.

Silent nociceptors are primary visceral afferents that have a particularly significant role in this process. They are normally inactive but become mechanosensitive and spontaneously active following tissue injury, creating a feedback loop that perpetuates visceral hypersensitivity [12]. This transformation is critical in the transition from acute nociceptive pain to chronic visceral hypersensitivity, implicating both peripheral and central mechanisms in the maintenance of pain. For instance, acute introduction of bile salts into the colon amplifies mechanosensitive colonic afferent firing during distension, leading to exaggerated pain responses and illustrating the interplay between chemical and mechanical sensitization [8].

Recent insights suggest that while acute injury often results in temporary mechanosensitization, persistent hyperalgesia may develop from prolonged or recurrent tissue damage. Animal models of colitis and visceral hypersensitivity demonstrate that colonic irritation can trigger long-lasting sensitization of the gut, even in the absence of ongoing inflammation or structural abnormalities [13]. Ongoing afferent input from peripheral sources leads to various pathological outcomes, including spontaneous motor abnormalities, hyperalgesia, pain, and allodynia [14]. Additionally, transient inflammation of the colon that leads to colonic distension can initiate sustained visceral hypersensitivity, with heightened abdominal muscle contractility and hyperexcitability of viscerosensitive neurons in the lumbosacral spinal cord (L6-S1) [9]. Agents such as lidocaine, which block sensitized visceral afferents, reverse these changes [14]. Significant alterations also occurred in the signaling pathways within the spinal cord, including the upregulation of pro-inflammatory mediators, which sustain pain processing even in the absence of ongoing inflammation in the gut [12,14]. Clinical observations in conditions such as complex regional pain syndrome further corroborate findings from these models [15]. In these cases, administration of peripherally applied anesthetics alleviates widespread pain and hypersensitivity, highlighting the role of nociceptive input in maintaining central sensitization.

Notably, continuous impulses from nociceptive colonic afferent neurons may partially sustain widespread zones of hypersensitivity observed in conditions such as fibromyalgia, neuropathic pain, and IBS. This hypothesis was evaluated in a double-blind crossover trial using intracolonic lidocaine jelly in IBS patients [16]. The trial showed significant reductions in hypersensitivity to nociceptive colonic distension and thermal stimuli applied to the foot, underscoring the role of tonic afferent input from the gut in secondary somatic hypersensitivity. Similar findings in animal models of IBS revealed that intracolonic lidocaine normalized both colonic and thermal hypersensitivity without detectable systemic lidocaine levels, indicating localized effects [13]. These findings underscore the contribution of persistent peripheral input from the gut to both primary visceral and secondary somatic hypersensitivity, as exemplified in IBS patients.

Beyond local tissue injury, systemic and developmental factors contribute to chronic hyperalgesia. For instance, neonatal colonic irritation in animal models leads to persistent visceral hypersensitivity and central sensitization, effects not observed in adults [13]. This observation suggests that there is a critical window during development when the nervous system is particularly susceptible to long-term alterations. Additionally, these findings align with clinical observations that approximately 25% of adults develop IBS following an enteric infection [7]. Transient inflammation of the small bowel or colon during such infections can result in prolonged gut sensitization, even after the resolution of active infection.

Afferent sensitization is often accompanied by central amplification, where spinal cord neurons receiving continuous input from sensitized visceral afferents become more responsive to incoming pain signals, exacerbating the perception of pain. The interplay between these peripheral and central processes creates a vicious cycle of hypersensitivity, where even mild stimuli can trigger intense pain [15]. This intricate relationship between the gut and the nervous system is central to the pathogenesis of a variety of chronic pain conditions, including IBS, interstitial cystitis, and ureteric colic [17]. In each of these conditions, the normal processing of sensory information is disrupted, leading to the perception of pain in response to stimuli that would typically be non-painful. The mechanisms underlying these disorders are multifactorial, involving both altered visceral afferent signaling and changes in central pain processing pathways.

#### 2.1.2. Central Sensitization and Viscerosomatic Convergence

Central sensitization, where spinal cord and brain neurons exhibit heightened excitability and hyperalgesic responses (increased pain sensitivity) to noxious stimuli, plays a central role in the development and persistence of chronic visceral pain [18,19]. This phenomenon amplifies and prolongs the perception of pain through increased glutamate release, N-methyl-D-aspartate (NMDA) receptor activation, and altered inhibitory signaling. Persistent activation of NMDA receptors and long-term potentiation (LTP) of synaptic transmission are critical components of spinal cord plasticity, promoting neuronal hyperexcitability and reinforcing synaptic strength [12,18,19]. Glial cell activation further exacerbates central sensitization. Microglia and astrocytes, activated by ongoing nociceptive input, release pro-inflammatory cytokines and chemokines (e.g., interleukin-1 beta (IL-1β), tumor necrosis factor-alpha (TNF-α)), perpetuating neuroinflammation and pain hypersensitivity [12]. This glial–neuronal crosstalk creates a self-reinforcing loop, contributing to the persistence of pain. Understanding these processes has informed therapeutic approaches targeting central sensitization, including receptor antagonists, ion channel blockers, and anti-inflammatory agents. In patients with IBS, this mechanism contributes to the heightened sensitivity to both visceral and somatic stimuli, suggesting that central sensitization is not merely a peripheral phenomenon but a systemic disorder [4].

Central sensitization is closely linked to viscerosomatic convergence, wherein nociceptive pathways from both the gut and somatic tissues share common spinal segments, particularly in the lumbosacral region. Experimental evidence has shown that sensory neurons in these regions can transmit pain signals originating from both visceral and somatic structures [4,17,18,19]. Studies further confirm that the overlap of these nociceptive pathways contributes to somatic hyperalgesia, where patients exhibit increased pain perception in areas innervated by the same spinal segments [2,15]. This overlap is thought to be responsible for the referred pain often reported by IBS patients whose discomfort extends beyond the gut to other parts of the body, such as the lower back or pelvis, and reflects the broader involvement of central mechanisms in IBS pathophysiology [20,21,22].

#### 2.1.3. Overlap with Chronic Pelvic Pain Disorders

The pathophysiology of chronic visceral pain is not restricted to the GI system; it frequently overlaps with other chronic pelvic pain disorders, including conditions like interstitial cystitis, pelvic floor dysfunction, and chronic pelvic pain syndrome [21]. Animal models have demonstrated that localized inflammatory injury in one visceral organ (e.g., the colon) can lead to sensitization of afferent pathways shared by other pelvic organs. This cross-sensitization between different pelvic organs, such as the bladder and intestines, provides a mechanistic basis for the clinical overlap of symptoms in patients with disorders like IBS and interstitial cystitis.

The concept of neural crosstalk, where afferent fibers from different organs converge at the spinal cord, reinforces the idea that chronic pelvic pain is a manifestation of a broader systemic disorder involving aberrant pain processing at both peripheral and central levels. Studies have further demonstrated that the primary visceral afferent pathways, which carry pain signals from the intestines, also contribute to altered sensations in other pelvic viscera (e.g., bladder) [2,8]. This suggests that chronic pelvic pain syndromes are likely to share common neural mechanisms that extend beyond the GI system.

### 2.2. Intestinal Barrier Dysfunction and Visceral Pain

**Figure 1:**
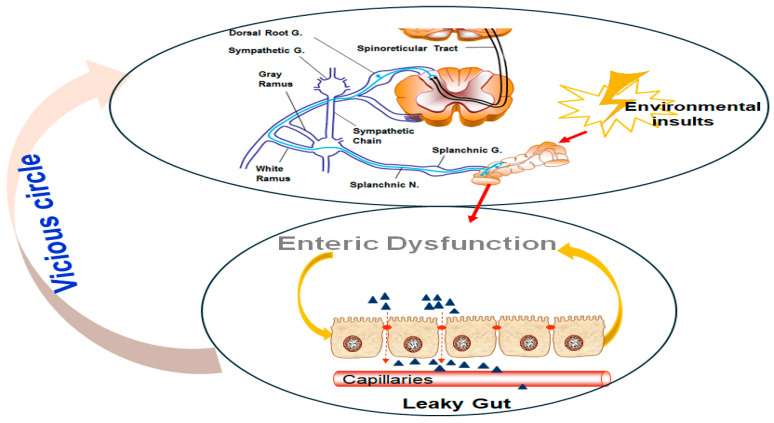
The vicious cycle of environmental insults, enteric dysfunction, and visceral hypersensitivity. (**Upper panel**) A neural pathway involving the splanchnic nerves and sympathetic chain, which respond to environmental insults, triggers nociceptive signaling. These signals are relayed through the DRG and spinoreticular tract, further exacerbating the stress response. (**Lower panel**) Enteric dysfunction is characterized by leaky gut syndrome, where compromised intestinal epithelial barrier integrity allows increased permeability to harmful molecules. This dysfunction amplifies systemic inflammation and sensitizes neural pathways, forming a feedback loop (vicious cycle) that perpetuates visceral hypersensitivity and chronic gut dysfunction.

In health, the GI tract serves as a barrier against harmful substances and supports nutrient absorption. However, disruptions in this barrier can lead to hyperpermeability, bacterial translocation, sepsis, and systemic inflammation, which have been implicated in conditions like celiac disease, inflammatory bowel disease, and IBS [6,7]. Post-infectious IBS (PI-IBS), particularly its diarrhea-predominant subtype (PI-IBS-D), is associated with chronic mucosal inflammation, elevated cytokine levels, and mast cell activation [10,16]. These factors increase intestinal permeability, allowing bacteria and antigens to penetrate the mucosal layer, triggering immune responses that exacerbate pain and diarrhea. This heightened permeability establishes a feedback loop wherein inflammatory mediators, bacteria, and antigens sustain afferent signaling to the spinal cord, potentially sensitizing spinal segments and perpetuating central sensitization (Figure 1). Targeting this interplay between intestinal permeability and spinal sensitization offers a promising avenue for treating PI-IBS-D and other disorders characterized by visceral pain [22].

Loss of the downregulated in adenoma (DRA; SLC26A3) protein compromises the intestinal epithelial barrier by reducing tight junction (TJ) and adherens junction proteins, such as ZO-1, occludin, and E-cadherin, thereby increasing colonic permeability [23]. DRA deficiency is associated with gut dysbiosis and microbial changes that partially affect TJ protein expression. Enhanced binding of the CUG triplet repeat RNA-binding protein 1 (CUGBP1) to occludin and E-cadherin genes in DRA knockout (KO) mice suggests posttranscriptional mechanisms contribute to barrier dysfunction and IBD pathogenesis [24,25].

Elevated vault RNA (vtRNA)1-1 levels, observed in EVs from shock patients and septic mice, impair intestinal epithelial renewal and barrier function by reducing intercellular junction proteins and Paneth cells. Mechanistically, vtRNA1-1 interacts with CUGBP1, increasing its association with mRNAs of Claudin-1, a TJ protein essential for maintaining intestinal barrier integrity, and occludin, thereby inhibiting their expression and exacerbating gut barrier dysfunction. These findings reveal a novel vtRNA1-1/CUGBP1 axis contributing to gut mucosal disruption during critical illness, providing insights into potential therapeutic targets for intestinal barrier restoration [26].

### 2.3. Epigenetic Mechanisms in Visceral Pain

Emerging evidence underscores the crucial role of epigenetic and non-coding RNA (ncRNA) mechanisms, particularly microRNAs (miRNAs), in the regulation of visceral pain. miRNAs are small, ncRNA molecules that fine-tune gene expression and affect a wide range of cellular processes by either degrading target messenger RNAs (mRNAs) or repressing their translation [27]. Through these mechanisms, miRNAs modulate critical cellular functions such as differentiation, proliferation, apoptosis, and neuroplasticity, which are essential for maintaining homeostasis in both the peripheral and central nervous systems.

miRNAs help regulate visceral pain through the molecular pathways that govern pain signaling and neuroplasticity. For instance, the miR-29 family, which is involved in physiological regulation processes, modulates enzymes that control DNA methylation, a key epigenetic modification that can alter gene expression. Through this regulation, miR-29 affects critical pathways involved in intestinal permeability, an essential factor in the development of chronic GI disorders. Increased intestinal permeability, often referred to as “leaky gut,” is a hallmark of conditions like IBS, where the gut barrier function is compromised, allowing noxious stimuli and inflammatory mediators to interact with sensory nerves and contribute to visceral hypersensitivity [28]. Additionally, in IBS-D, upregulated miR-29a/b levels have been linked to reduced expression of Claudin-1 [29]. This downregulation contributes to increased permeability and chronic hypersensitivity. Silencing the miR-29 cluster has been shown to reverse intestinal hyperpermeability, indicating that miR-29 represents a potential therapeutic target.

The circular RNA (circRNA) Cdr1as acts as a repressor of intestinal epithelial regeneration and defense, with levels increasing in conditions such as colitis and sepsis in both mice and humans. Ablation of Cdr1as enhances intestinal mucosal renewal, promotes injury-induced epithelial regeneration, and provides protection against colitis. The inhibitory effects of Cdr1as on epithelial repair are mediated, at least partially, through interactions with miR-195, highlighting its role in impaired mucosal renewal [30].

IL-1β increases intestinal permeability by upregulating the miRNA MIR200C-3p, which suppresses occludin expression in enterocytes, disrupting the TJ barrier. In mice and patients with colitis, elevated IL-1β and MIR200C-3p levels correlate with reduced occludin and increased TJ permeability, a hallmark of intestinal inflammation. Targeting MIR200C-3p with an antagonist preserves occludin expression, reduces permeability, and mitigates barrier dysfunction, offering potential therapeutic benefits for colitis [31].

miRNAs also play a significant role in the regulation of pain pathways within the CNS. miRNAs can modulate the expression of genes involved in neuroplasticity, the ability of the nervous system to reorganize itself in response to injury or environmental changes [27]. In the context of chronic visceral pain, altered miRNA expression may contribute to the sensitization of spinal cord neurons, amplifying pain signals and promoting the development of central sensitization in chronic pain disorders, and can lead to the persistence of pain long after the initial injury or inflammation has subsided. Moreover, miRNAs can also influence the inflammatory milieu within the gut and CNS. By regulating the expression of cytokines, chemokines, and other inflammatory mediators, miRNAs contribute to the modulation of the immune response, which is often dysregulated in individuals with chronic visceral pain [10]. This dysregulation may exacerbate pain and prolong the sensitization of both peripheral and central pain pathways. miRNAs also play a role in modulating nociception and pain thresholds. For instance, miR-328 and miR-320 downregulate the neurokinin-1 receptor (NK1R) in bladder pain syndrome, while let-7 miRNAs influence opioid tolerance by targeting mu-opioid receptors [32,33].

The complexity of miRNA regulation suggests that these molecules could serve as both biomarkers for diagnosing chronic visceral pain disorders and therapeutic targets for novel interventions [33]. By selectively modulating specific miRNAs, it may be possible to restore normal gene expression patterns and alleviate the persistent pain associated with conditions such as IBS, interstitial cystitis, and other FGIDs.

miRNAs thus represent a crucial layer of regulation in the pathophysiology of chronic visceral pain. Their ability to influence gene expression and cellular processes, particularly in the context of intestinal permeability, pain signaling, and neuroplasticity, positions them as key players in the development and persistence of visceral hypersensitivity. Further research into the specific roles of individual miRNAs in these pathways may provide valuable insights into the molecular mechanisms underlying chronic visceral pain and open up new avenues for targeted therapeutic strategies.

## 3. Key Molecular Mediators and Possible Targets for Treatment of Visceral Pain

Current treatments for chronic visceral pain have limited efficacy [7], which highlights the pressing need for a deeper understanding of its underlying mechanisms. Insights into primary afferent physiology, neural crosstalk, and the integration of peripheral and central pain pathways hold significant promise for therapeutic innovation. Addressing the multifaceted nature of visceral pain through comprehensive research can pave the way for novel treatments, ultimately improving outcomes for patients with IBS and other DGBIs.

Recent advancements have identified potential therapeutic targets, such as specific ion channels, receptors, and signaling molecules that mediate visceral hypersensitivity [10]. By modulating these pathways, it may be possible to develop more effective treatments for chronic visceral pain, aimed not only at alleviating symptoms but also at addressing the underlying mechanisms of pain generation and maintenance. Furthermore, the growing understanding of the role of the CNS in amplifying visceral pain suggests that treatments targeting central sensitization, such as neuromodulatory therapies, may offer promise for patients suffering from conditions like IBS. In addition, approaches aimed at restoring gut barrier function or modulating the gut microbiota may hold potential for preventing or reversing the peripheral sensitization of visceral afferents.

### 3.1. Neurotransmitters and Neuromodulators in Pain Signaling

#### 3.1.1. Glutamate and Gamma-Aminobutyric Acid (GABA)

Glutamate and GABA are fundamental to excitatory and inhibitory signaling in the CNS, particularly in pain modulation [15]. Glutamate, the primary excitatory neurotransmitter, is crucial for transmitting nociceptive signals. It works through ionotropic receptors, such as the NMDA and α-amino-3-hydroxy-5-methyl-4-isoxazolepropionic acid (AMPA) receptors, which mediate rapid synaptic transmission, and metabotropic glutamate receptors (mGluRs), which regulate synaptic plasticity. Enhanced glutamatergic signaling in the spinal cord is vital for central sensitization, a process characterized by heightened nociceptive neuron responsiveness. This phenomenon, involving sustained activity-dependent synaptic changes, including phosphorylation of NMDA receptors and increased AMPA receptor trafficking, has been shown to amplify excitatory signaling [14]. These mechanisms reflect pathological neural plasticity in response to persistent nociceptive inputs, thereby contributing to visceral hypersensitivity.

In contrast, GABA serves as the principal inhibitory neurotransmitter that maintains neural homeostasis by counterbalancing excitatory signals. GABAergic inhibition occurs through GABA-A (ionotropic) and GABA-B (metabotropic) receptors that suppress nociceptive transmission by hyperpolarizing postsynaptic neurons and reducing neurotransmitter release. Dysfunction in GABAergic pathways, such as downregulated receptor expression or altered chloride homeostasis, leads to increased pain sensitivity. GABAergic neurons in the ventral spinal cord are also targets for μ-opioid receptor-mediated presynaptic inhibition, indicating that GABA signaling and analgesic mechanisms interact [15].

These disruptions likely contribute to conditions like IBS, where impaired inhibitory control heightens pain perception. Together, glutamate-driven excitation and GABA-mediated inhibition are key regulators of nociceptive signaling and represent therapeutic targets for chronic pain management.

#### 3.1.2. Substance P and Calcitonin Gene-Related Peptide (CGRP)

In nociceptive signaling, substance P is a critical neuropeptide that is produced in response to noxious stimuli [34,35,36]. Released by primary sensory neurons, it binds to neurokinin-1 (NK1) receptors to amplify pain transmission. Substance P also mediates neurogenic inflammation, contributing to the sensitization associated with chronic pain. By facilitating communication between the peripheral and central nervous systems, substance P plays a dual role in pain propagation and inflammation, making it a key target in managing visceral pain syndromes.

CGRP, often co-released with substance P, complements its effects by targeting CGRP receptors. It induces vasodilation and promotes inflammatory responses, integral to both peripheral and central sensitization. CGRP’s role in migraine-related visceral symptoms and GI pain disorders underscores its importance in the vascular-neural interplay underlying visceral pain. CGRP and substance P can also combine synergistically to contribute to pain and inflammation [34,35,36]. These insights support their potential as therapeutic targets for visceral pain management.

#### 3.1.3. Serotonin (5-HT): Receptor Subtypes

Serotonin (5-HT) is a key regulator of GI function, with receptor subtypes playing distinct roles in physiological and pathological processes [36,37]. 5-HT3 receptors are ligand-gated ion channels that mediate rapid excitatory neurotransmission. Their dysregulation contributes to visceral hypersensitivity in GI pain conditions like IBS. Therapeutic use of 5-HT3 antagonists has proven effective in alleviating pain and discomfort in DGBIs. Conversely, 5-HT4 receptors, part of the G-protein-coupled receptor family, regulate GI motility and sensitivity. Agonists targeting 5-HT4 receptors improve gut motility and reduce pain, highlighting their therapeutic potential.

Dysregulated serotonin signaling is a hallmark of visceral pain syndromes, linked to hypersensitivity and altered gut motility. Serotonin has a multifaceted role in GI physiology and pathology, providing a foundation for targeted drug development. Modulating serotonin signaling may be a promising approach to improve symptoms and quality of life in DGBIs [36,37].

#### 3.1.4. Transient Receptor Potential Channels (TRP)

TRPV1 is a nonselective cation channel crucial for detecting noxious stimuli such as extreme heat, acidity, and capsaicin. Expressed primarily in peripheral nociceptive neurons, TRPV1 contributes to inflammatory pain responses and hyperalgesia by lowering its activation threshold during inflammation. TRPV1’s role in pain pathways has been well defined, suggesting it as a possible target for therapies aimed at alleviating visceral pain [38,39].

Similarly, environmental irritants (e.g., mustard oil) and endogenous inflammatory mediators activate the Transient Receptor Potential Ankyrin 1 (TRPA1) channel, which contributes to inflammatory hyperalgesia. TRPA1 antagonists reduce pain behaviors in animal models, offering novel strategies for managing visceral pain. TRPV1 and TRPA1 have a dynamic interplay, playing complementary roles in pain processing [38].

#### 3.1.5. Voltage-Gated Sodium Channels

Voltage-gated sodium channels (NaV), particularly NaV1.7 and NaV1.8, are pivotal in neuronal excitability and pain signaling [40]. These channels, predominantly expressed in sensory neurons, facilitate action potential propagation. Pathological conditions like tissue injury or inflammation enhance NaV channel activity, resulting in neuronal hyperexcitability and chronic pain syndromes. NaV1.1 is involved in mechanical pain, as shown by the use of selective spider toxin inhibitors that significantly reduce pain responses [41]. These findings underscore NaV channels as key targets for pain management.

#### 3.1.6. Catechol-O-Methyltransferase (COMT)

COMT modulates nociceptive signaling by degrading catecholamines such as dopamine, epinephrine, and norepinephrine. Genetic variations in COMT expression influence pain perception and susceptibility to chronic pain conditions such as fibromyalgia and migraines [42]. The V158M single-nucleotide polymorphism (SNP) in the COMT gene has been linked to heightened pain sensitivity, underscoring its role in individual pain variability [43].

**Figure 2:**
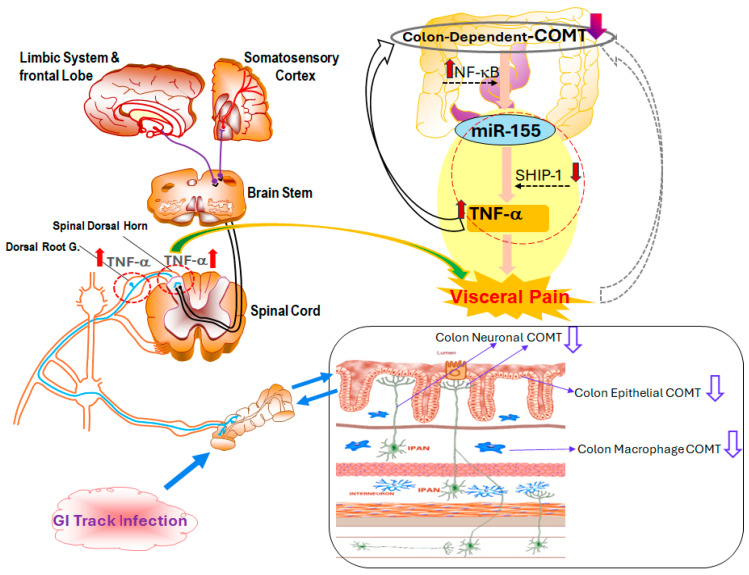
Mechanistic overview of colon-dependent catechol-O-methyltransferase (COMT) and its role in visceral pain. Both central and peripheral pathways are involved in visceral pain following GI tract infection. Gut barrier dysfunction, immune activation, and neural hypersensitivity all contribute to the pathogenesis of visceral pain. In epithelial, neuronal, and macrophage cells, colon-dependent COMT expression is reduced, resulting in dysregulated inflammatory responses. Upregulation of nuclear factor kappa-light-chain-enhancer of activated B cells (NF-κB) and miR-155, coupled with the suppression of Src homology 2 domain-containing inositol phosphatase 1 (SHIP-1), leads to increased TNF-α production, amplifying nociceptive signaling. Pro-inflammatory cytokines such as TNF-α activate NF-κB pathways within DRG and spinal dorsal horn neurons, further sensitizing pain pathways and transmitting signals to the brain stem, limbic system, and somatosensory cortex.

Recent studies have shown that loss or reduced expression of COMT not only affects catecholamine metabolism but also contributes to increased production of pro-inflammatory cytokines, particularly TNF-α, via a downstream miRNA-dependent mechanism. Specifically, decreased COMT activity leads to upregulation of miR-155, which in turn promotes TNF-α expression in enteric neurons and macrophages [44] (Figure 2). TNF-α then acts on nociceptive pathways to sensitize colonic and dorsal root ganglion (DRG) neurons, thereby enhancing visceral pain signaling [45].

This COMT-miR-155-TNF-α axis provides a mechanistic bridge between genetic susceptibility and immune-driven inflammation in post-infectious IBS with diarrhea (PI-IBS-D), offering a potential therapeutic target for interrupting the cycle of chronic abdominal pain.

#### 3.1.7. Ion Channels

In addition to key mediators of visceral pain pathophysiology, such as cytokines, prostaglandins, and neuropeptides, which are critical for activating and sensitizing nociceptors [39], ion channels like transient receptor potential vanilloid 1 (TRPV1) and acid-sensing ion channels (ASICs) are important for detecting noxious stimuli [35]. Because altered expression and function of these channels have been strongly implicated in the development of visceral hypersensitivity [38,39], these molecular mediators may be potential targets for therapeutic intervention. Furthermore, targeted therapies aimed at modulating the activity of specific ion channels and receptors involved in nociception could provide substantial benefits over traditional analgesics, including enhanced efficacy with fewer side effects. For example, TRPV1 antagonists and ASIC inhibitors are being explored as potential options for mitigating visceral pain by reducing afferent sensitization [38].

In particular, visceral hypersensitivity involving TRPV1-mediated nociceptive signaling and inflammation is a key mechanism underlying abdominal pain in patients with IBS with constipation (IBS-C). Tenapanor, a small-molecule inhibitor of the sodium/hydrogen exchanger isoform 3 (NHE3), inhibits absorption of sodium and phosphate in the GI tract. Treatment with tenapanor improves GI motility, decreases intestinal permeability and inflammation, and normalizes TRPV1 signaling, which may collectively reduce visceral hypersensitivity and associated abdominal pain. These findings suggest that targeting visceral hypersensitivity could be a promising approach for alleviating abdominal pain in IBS-C patients [46,47].

Table 1 provides a comprehensive overview of key neurotransmitters and neuromodulators involved in pain signaling.

The dual involvement of peripheral and central mechanisms in chronic visceral hypersensitivity underscores the complexity of these conditions and highlights the need for a multifaceted therapeutic approach. As research into the underlying mechanisms of visceral pain continues to evolve, there is increasing hope that novel interventions targeting both peripheral and central pathways will offer relief to patients suffering from these debilitating disorders, ultimately improving their quality of life.

## 4. Conclusions

Chronic visceral pain, particularly in the context of disorders such as IBS, is a complex, multifactorial condition that involves both peripheral and central mechanisms. Several well-supported mechanisms have been implicated in the development and persistence of visceral pain and hypersensitivity, including (i) nociceptive input from the colon, which contributes to the induction and maintenance of hypersensitivity; (ii) increased intestinal permeability, driving or sustaining visceral nociceptive responses; and (iii) alterations in miRNAs and EVs within target tissues, which may influence local and systemic signaling pathways. The interplay of these factors emphasizes the need for an integrated approach to understanding and treating this pervasive condition.

Recent advancements suggest that epigenetic regulation plays a pivotal role in modulating stress-induced visceral pain. The pathophysiology of GI disorders, including IBS, is increasingly linked to aberrant expression of microRNAs (miRNAs) and other molecular mechanisms. Emerging models also highlight the involvement of DNA methylation and disrupted miRNA signaling pathways as key contributors to these processes. Despite these insights, the molecular basis and neurobiology of specific patient endophenotypes experiencing visceral pain remain incompletely understood, underscoring the need for further research to identify actionable therapeutic targets.

While these mechanisms offer valuable insights, unidentified pathways likely contribute to the complex interplay between peripheral and central processes in chronic visceral pain. Synergistic interactions among these mechanisms, along with transient physiological triggers such as gut inflammation or increased intestinal permeability, may initiate and perpetuate visceral hypersensitivity.

Future studies should aim to unravel these intricate interactions to provide a more comprehensive understanding of visceral nociception. Such efforts could pave the way for novel, precisely targeted therapies that improve outcomes for patients with chronic visceral pain, surpassing the efficacy of current treatment options. By integrating insights from molecular biology, neurobiology, and patient-specific endophenotypes, we can move toward more effective therapies that address not only the peripheral origins of pain but also the central mechanisms that perpetuate it to make significant strides in the management of chronic GI disorders.

## Figures and Tables

**Table 1:** Neurotransmitters and Neuromodulators in Pain Signaling: Roles, Mechanisms, and Therapeutic Implications.

Neurotransmitter/ Neuromodulator	Mechanism	Role in Pain Signaling	Therapeutic Implications
Glutamate [22]	Primary excitatory neurotransmitter in the CNS; acts through NMDA, AMPA, and mGluRs	Mediates excitatory nociceptive signaling, contributing to central sensitization	Targeting glutamate receptors (NMDA, AMPA) and GABA receptors can help manage chronic pain and hypersensitivity, particularly in IBS
GABA [15]	Principal inhibitory neurotransmitter; acts through GABA-A and GABA-B receptors	Maintains neural homeostasis by inhibiting excessive excitatory signaling	
Substance P [36]	Released by primary sensory neurons; binding to NK1 receptors amplifies pain signals and mediates inflammation	Enhance pain transmission and contribute to neurogenic inflammation, facilitating peripheral and central sensitization	Potential targets for managing visceral pain syndromes, especially in IBS and related disorders
CGRP [35]	Promotes vasodilation and inflammation, often co-released with substance P		
Serotonin (5-HT) [36,37]	5-HT3 receptors: mediate excitatory transmission and contribute to visceral pain	5-HT signaling regulates gut function and is implicated in conditions like IBS Dysregulated 5-HT3 signaling contributes to visceral hypersensitivity	Modulation of serotonin receptors, particularly 5-HT3 antagonists and 5- HT4 agonists, offers potential therapeutic strategies for gut disorders
	5-HT4 receptors: regulate GI motility and sensitivity	5-HT signaling regulates gut function and is implicated in conditions like IBS 5-HT4 agonists improve motility and reduce pain	
TRPV1 channels [38,39]	Activated by noxious stimuli, such as heat and acid	Contribute to inflammatory pain responses and hyperalgesia Lowers its activation threshold during inflammation	Antagonists show promise as novel therapies for visceral pain and hyperalgesia, particularly in inflammatory conditions
TRPA1 channels [38]	Activated by environmental irritants and inflammatory mediators	Contribute to inflammatory pain responses and hyperalgesia participates in inflammatory pain	
Voltage-gated sodium channels [40,41]	NaV1.7 and NaV1.8 channels facilitate action potential propagation in sensory neurons, with activity enhanced in pathological conditions like inflammation	Essential for pain signaling and contribute to neuronal hyperexcitability in chronic pain conditions	NaV channel blockers are potential pain management therapies, particularly for neuropathic and inflammatory pain
COMT [43,44]	COMT degrades catecholamines (dopamine, norepinephrine), modulating pain sensitivity	Genetic variants in COMT influence pain perception; higher COMT activity is associated with reduced pain sensitivity Inflammation can modulate COMT’s effects on pain signaling	Targeting COMT in combination with other therapies may reduce chronic pain, particularly in conditions like fibromyalgia and IBS

## Data Availability

No new data were created or analyzed in this study.

## Abbreviations

The following abbreviations are used in this manuscript:

ASIC	Acid-sensing ion channel
CGRP	Calcitonin gene-related peptide
circRNA	Circular ribonucleic acid
CNS	Central nervous system
COMT	Catechol-O-methyltransferase
CUGBP1	CUG triplet repeat RNA-binding protein 1
DGBI	Disorder of gut–brain interaction
DRA	Downregulated in adenoma protein
DRG	Dorsal root ganglia
EV	Extracellular vesicle
FGF	Fibroblast growth factor
FGID	Functional gastrointestinal disorder
GABA	Gamma-aminobutyric acid
GI	Gastrointestinal
HIF-1α	Hypoxia-inducible factor 1 subunit alpha
IBS	Irritable bowel syndrome
IBS-C	IBS with constipation
IL	Interleukin
lncRNA	Long non-coding RNA
IJ	Intercellular junction
KO	Knockout
LTP	Long-term potentiation
miRNA	MicroRNA
mRNA	Messenger RNA
ncRNA	Non-coding RNA
NF-κB	Nuclear factor kappa-light-chain-enhancer of activated B cells
NHE3	Sodium/hydrogen exchanger isoform 3
NMDA	N-methyl-D-aspartate
NO	Nitric oxide
PAMPs	Pathogen-associated molecular patterns
PI3K	Phosphatidylinositol 3 kinase
PI-IBS	Post-infectious IBS
PI-IBS-C	PI-IBS with constipation
PI-IBS-D	PI-IBS with diarrhea
ROS	Reactive oxygen species
SHIP-1	Src homology 2 domain-containing inositol phosphatase 1
TGF-β	Transforming growth factor beta
TJ	Tight junction
TLR	Toll-like receptor
TNF-α	Tumor necrosis factor-alpha
TRPA1	Transient receptor potential ankyrin 1
TRPV1	Transient receptor potential vanilloid 1
vtRNA	Vault RNA
